# Role of the Intersections of Gender, Race and Sexual Orientation in the Association between Substance Use Behaviors and Sexually Transmitted Infections in a National Sample of Adults with Recent Criminal Legal Involvement

**DOI:** 10.3390/ijerph19074100

**Published:** 2022-03-30

**Authors:** Tyler D. Harvey, Ijeoma Opara, Emily A. Wang

**Affiliations:** 1 SEICHE Center for Health and Justice, Yale School of Medicine, New Haven, CT 06511, USA; emily.wang@yale.edu; 2 Department of Social and Behavioral Sciences, Yale School of Public Health, New Haven, CT 06511, USA; ijeoma.opara@yale.edu; 3 Department of Internal Medicine, Yale School of Medicine, New Haven, CT 06511, USA

**Keywords:** substance use, sexually transmitted infection, incarceration, intersectionality

## Abstract

Limited research has focused on how substance use and sexual risk behaviors differ among individuals impacted by the criminal legal system based on social identities. Using the National Survey on Drug Use and Health, we estimated relative risk for reporting a sexually transmitted infection (STI) among intersectional social groups with criminal legal involvement using a modified Poisson regression. We then utilized multivariate logistic regression and marginal effects to measure associations between substance use behaviors and STIs and to estimate whether these varied among the intersectional social groups with elevated STI rates. Three groups had elevated risk of reporting an STI compared to white, heterosexual men: white, heterosexual women (1.53, 95% CI: 1.05–2.20); Black, heterosexual women (2.03, 95% CI: 1.18–3.49); and white, gay or bisexual men (5.65, 95% CI: 2.61–12.20). Considering the intersections of gender, race, and sexual orientation, elevated risks for STIs among white and Black heterosexual women were mitigated after adjusting for substance use alongside other confounders. Only those who identified as white, gay or bisexual, and male had increased STI risk after controlling for substance use. Interventions targeting Black and white heterosexual women’s sexual health following incarceration should focus on substance use and interventions targeting white, gay or bisexual men should focus on healthy sexual behaviors, HIV/STI screening, and care continuum efforts.

## 1. Introduction

Each year in the United States (U.S.), 11 million people experience incarceration, with an additional 4.4 million people living under a form of community corrections control [1,2]. Incarceration disproportionately impacts individuals, families, and communities experiencing marginalization in society, such as racial and sexual minorities. One in three Black men will go to prison in their lifetimes [3]. Black women are incarcerated at twice the rate of white women, and one in two Black transgender individuals have been incarcerated [4,5]. Lesbian, gay, and bisexual individuals are incarcerated at three times the rate of the general population [6]. Populations with marginalized identities leaving incarceration have unique needs, which are often unmet given the focus of reentry programs on meeting the needs of heterosexual, male populations [7,8,9,10].

Incarceration is associated with heightened risk of morbidity, including an increased likelihood of acquiring a sexually-transmitted infection (STI) [11,12,13]. People with incarceration histories have higher rates of STIs compared to the general population [14,15]. A study of 247,211 individuals showed those with incarceration histories were 3.9 times more likely to have chlamydia, 6.6 times more likely to have human immunodeficiency virus (HIV), and 3.6 times more likely to have syphilis compared to those never incarcerated [14]. Having an incarceration history is associated with higher rates of risky sexual behaviors, such as engaging in transactional sex, having condomless sex, and substance use during sex [11,13,16,17,18,19,20,21]. A study of 532 HIV-positive men found recent incarceration to be associated with condomless sex related to drug use (adjusted odds ratio: 6.53, 95% confidence interval (CI): 2.18–19.52) [19]. Another study of 552 young men in the New York City jail system found that alcohol and marijuana use prior to incarceration was related to STI/HIV risk behaviors, such as having multiple partners [21].

No national study has examined the association between substance use and STIs among populations with recent criminal legal system involvement and especially how an individual’s social identities (e.g., race and gender) may impact this association [14,15,22,23,24,25,26,27]. However, previous work has highlighted differences in sexually risky behaviors by a single social identity among those impacted by incarceration. Women with incarceration histories are 2.8 times more likely to have HIV than men who have been incarcerated; this is nearly seven times higher for Black individuals than white individuals [14]. Research considering multiple identities is less common, but recent studies show that among justice involved individuals, Black men who have sex with men and Black heterosexual women have worse sexual health [19,28,29]. For instance, in a study of 1553 Black men who have sex with men, men who have been incarcerated had an increased rate of chlamydia compared to those never incarcerated (adjusted prevalence ratio: 1.47, 95% CI: 0.98–2.20) [30]. Black women have been shown to have a hazard ratio of 6.02 (95% CI: 3.36–10.80) for testing positive for syphilis in the first year following release from jail compared to white women [31]. 

Given that mass incarceration disproportionately impacts individuals along the lines of gender, race, and sexual orientation, understanding the role of these intersecting identities in shaping sexual health is critical. Intersectionality theory, originally rooted in Black feminist scholarship, acknowledges how multiple marginalized identities do not operate in silos but in conjunction to inform individual experiences of health and that interlocking structural systems of power influence the health of marginalized groups [32,33]. For this study, we aimed to: (1) estimate the age-adjusted relative risk of reporting an STI among individuals with recent criminal justice involvement using intersectional social identifiers and (2) examine the association between substance use behaviors and STIs among subgroups with elevated STI risk. Such findings will inform both clinical care and interventional work to foster healthy substance use and sexual health in this high-risk population.

## 2. Methods

### 2.1. Data Source

We used publicly available data from the National Survey on Drug Use and Health (NSDUH), a nationally representative, cross-sectional survey of noninstitutionalized individuals over the age of 12 years old [34]. The NSDUH annually surveys approximately 70,000 individuals across the U.S. with a multi-stage area probability approach. We pooled data from years 2015–2018 and limited our sample to adults aged 18–64 years old. Our use of publicly available, deidentified data exempted this study from review according to Yale University Institutional Review Board policies. 

### 2.2. Measures

#### 2.2.1. Recent Criminal-Legal Involvement 

Our sample consisted of those who had been arrested and those who had spent time on parole or probation in the past 12 months. Respondents were asked “not counting minor traffic violations, have you ever been arrested and booked for breaking the law?” and then “not counting minor traffic violations, how many times during the past 12 months have you been arrested and booked for breaking a law?”. The NSDUH also asked “were you on probation at any time during the past 12 months” and “were you on parole, supervised release, or other conditional release from prison at any time during the past 12 months?”. Parole and probation are types of community correctional supervision in which individuals are allowed to serve part of their incarceration within the community instead of in prison. We excluded those never arrested, those not on probation or parole in the past 12 months, and those who had been arrested more than 12 months ago. 

#### 2.2.2. Social Identities Variables 

While many social identifiers may be associated with sexual risk and substance use behaviors among individuals with criminal legal involvement (e.g., age), we focused this analysis on only three social identities—race, gender, and sexual orientation—given previous research on STIs disproportionately impacting individuals along these lines [35,36]. Because the lives of Black individuals in the U.S. are defined by structural inequalities, including mass incarceration, housing discrimination, and unequal access to quality healthcare, that contribute to poor health outcomes, we examined the intersections of race in addition to gender and sexual orientation [37]. The NSDUH recodes race and ethnicity into one variable with the following categories: “Non-Hispanic, White”, “Non-Hispanic, Black”, “Non-Hispanic, Native American/Alaskan Native”, “Non-Hispanic, Native Hawaiian/Other Pacific Islander”, “Non-Hispanic Asian”, “Non-Hispanic, More than One Race”, and “Hispanic”. We delimited the sample to only include two racial groups (white, non-Hispanic and Black, non-Hispanic). Respondents were given two options for reporting gender (“male” and “female”). Respondents were asked “which of the following do you consider yourself to be?” and presented the following options: “Heterosexual, that is straight,”, “Lesbian or Gay”, “Bisexual”, “Don’t Know”, and “Refused”. Sexual orientation was categorized as heterosexual and gay/bisexual; we grouped gay and bisexual individuals together to avoid sample size and power issues. We then created a variable at the intersection of all three identities: heterosexual, white men; heterosexual, white women; heterosexual, Black men; heterosexual, Black women; gay/bisexual, white men; gay/bisexual, white women; gay/bisexual, Black men; and gay/bisexual, Black women. 

#### 2.2.3. Substance Use Behaviors 

Our primary independent variables were (1) any illicit drug use, (2) any illicit drug use disorder (not including alcohol use disorder but including all other substance use disorders including marijuana), (3) any illicit drug or alcohol use disorder, and (4) any polysubstance use. Polysubstance use was categorized as reporting any usage of two or more substances, including alcohol, marijuana, cocaine, heroin, hallucinogens, inhalants, and methamphetamine, included in this study in the past 12 months. All substance use variables were binary “yes/no” variables. 

For our secondary outcomes, we utilized substance use behaviors among different drugs. For alcohol use, we examined binge alcohol use in the past 30 days, heavy alcohol use in the past 30 days, and alcohol use disorder. Binge alcohol use was assessed by asking “During the past 30 days, that is since [DATEFILL], on how many days did you have [4 or more]/[5 or more] drinks on the same occasion?” and was defined as responding with at least 1 day. Heavy alcohol use was defined as drinking five or more drinks for males or four or more drinks for females on the same occasion for 5 or more days in the past 30 days. We examined use of the following substances and whether their use met criteria to be diagnosed as a disorder: marijuana, cocaine, heroin, hallucinogens, inhalants, and methamphetamine. For crack cocaine, we only had information regarding whether an individual reported any usage of it. Respondents were asked “how long has it been since you lasted used [marijuana or hashish]/[cocaine]/[crack]/[heroin]/[hallucinogens]/[inhalants]/[methamphetamine]?” with response options being “Within past 30 days”, “More than 30 days but within past 12 months”, “More than 12 months ago”, “Never”, and “Refused”. For each substance, we created a binary variable indicating whether the individual reported using that substance “Within past 30 days” and “More than 30 days but within past 12 months”. 

All substance use behaviors were reflective of the previous 12-month period, unless otherwise noted, and the NSDUH classifies substance use disorders in accordance with the Diagnostic and Statistical Manual of Mental Disorders, 4th edition (DSM IV) [38]. 

#### 2.2.4. Sexually Transmitted Infection Outcome

Our primary dependent variable was a recent self-reported STI, which was assessed with the question: “During the past 12 months, did you have a sexually transmitted disease, such as chlamydia, gonorrhea, herpes, or syphilis?” with response options of “Yes”, “No”, “Don’t Know”, “Refused”, and “No Answer”. A binary variable was created for those respondents who selected “Yes” and “No”. 

#### 2.2.5. Other Sociodemographic and Health Covariates

Age was the only confounder in our analysis on relative risk given previous research on the increased risk of STI among young adults [39]. This is the recommended approach when describing intersectional health disparities to avoid over-adjustment bias because other factors, such as income, are along the casual pathway between intersecting identities of gender, race, and sexual orientation and STI risk [40,41]. In our analyses of the association between substance use behaviors and reported STI, we examined several sociodemographic characteristics, including age, gender, race and ethnicity, sexual orientation, education level, poverty status, metropolitan status, marital status, and health care utilization. Metropolitan status was defined by categorizing rural–urban continuum codes into large metropolitan, small metropolitan, and nonmetropolitan areas [42]. Health care utilization was assessed by the number of times an individual had reported visiting a doctor’s office, clinic, or some other place to discuss their own health in the past 12 months. 

### 2.3. Statistical Analysis 

We utilized descriptive statistics to detail sociodemographic information and health behaviors of our sample of individuals with criminal legal involvement in the past 12 months. We approximated relative risk of reporting an STI and corresponding 95% confidence intervals at the intersections of race, gender, and sexual orientation with modified Poisson regression models [43]. Abiding by quantitative intersectional research guidelines, we selected the group with the most privileged identities as the reference group to estimate relative risk (i.e., white, heterosexual men) [41]. We then used logistic regression and estimated average marginal effects of the associations on how substance use behaviors affect the probability of reporting an STI while holding all other variables constant in the full sample and then within groups identified to have heightened relative risk of acquiring an STI [44]. For estimating average marginal effect, we excluded individuals who had missing data for the covariates in our models, which was less than 4% of respondents, to take advantage of all covariate information. All analyses were implemented using the complex NSDUH survey weighting, which allows for nationally representative estimates. The statistically significant threshold was *p* < 0.05, and all tests were two-sided. Our analyses were conducted with R [45].

## 3. Results

Our sample included 7260 individuals involved with the criminal legal system in the past 12 months, representative of approximately 7,205,119 adults between 2015–2018; 19.6% (95% CI: 18.3–21.0%) were on parole, 54.7% (95% CI: 53.0–56.3%) were on probation, and 69.1% had been arrested (95% CI: 67.8–70.5%). Thirty percent (95% CI: 28.4–31.5%) were women; 21.4% were non-Hispanic, Black (95% CI: 20.0–22.8%); and 8.6% identified as gay or bisexual (95% CI: 7.5–9.7%). Nearly one in three reported living in poverty (33.2%, 95% CI: 31.4–35.0%). The majority of respondents reported having health insurance (73.6%, 95% CI: 72.5–74.7%) (Table 1).

**Figure 1:**
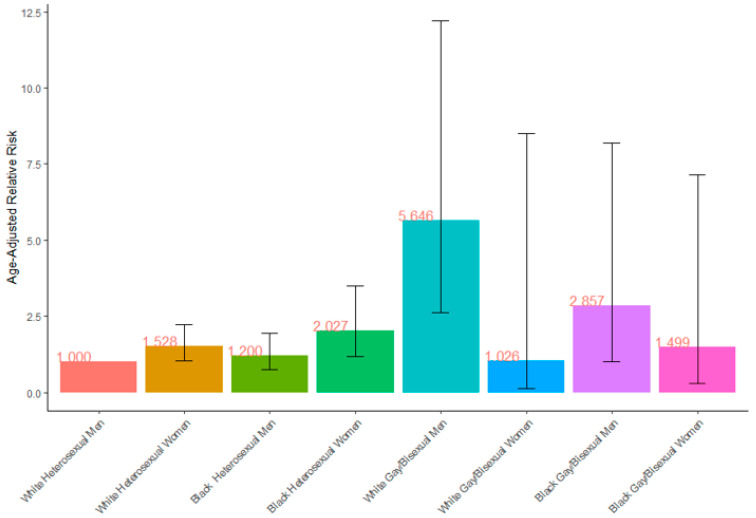
Age-adjusted relative risk of reporting a sexually transmitted infection in past 12 months among a national sample of individuals with recent criminal legal involvement by gender, race, sexual orientation, and their intersections. (N = 4530). Error bars represent 95% confidence intervals of estimates.

Among the weighted sample, 5.2% of individuals with recent criminal legal involvement reported an STI in the past 12 months (95% CI: 4.4–5.9%). Along the intersections of race, gender, and sexual orientation, the following had significantly higher age-adjusted relative risks for reporting an STI compared to white, heterosexual men: white, heterosexual women (1.53, 95% CI: 1.05–2.2); Black, heterosexual women (2.03, 95% CI: 1.18–3.49); and white, gay/bisexual men (5.65, 95% CI: 2.61–12.20) (Figure 1).

After adjusting for sociodemographic characteristics and health care utilization, substance use behaviors were associated with reporting an STI (Table 2). Particularly, heavy alcohol use (2.4%, 95% CI: 0.3–4.4%), binge alcohol use (2.2%, 95% CI: 0.8–3.7%), and alcohol use disorder (2.8%, 95% CI: 0.2–5.5%) increased the probability of a recent STI. Reporting any use of marijuana (3.0%, 95% CI: 1.6–4.3%), cocaine (3.2%, 95% CI: 0.6–5.7%), hallucinogen (4.1%, 95% CI: 0.8–7.4%), and methamphetamine (3.7%, 95% CI: 0.7–6.9%) was also associated with increased likelihood of reporting a recent STI.

Along the intersections of gender, race, and sexual orientation, we found that only white, gay/bisexual men had a significantly increased probability of reporting an STI after controlling for illicit drug use (16.2%, 95% CI: 2.3–30.2%), illicit drug use disorder (16.7%, 95% CI: 2.9–30.5%), illicit drug or alcohol use disorder (16.8%, 95% CI: 2.9–30.7%) or polysubstance use (16.0%, 95% CI: 2.1–29.9%) compared to white, heterosexual men (Table 3). Age was negatively associated with reporting a recent STI, and healthcare utilization was positively associated with reporting a recent STI when controlling for overall drug use.

## 4. Discussion

Our findings contribute to a growing literature in understanding how health behaviors and outcomes among individuals with criminal legal involvement vary along the intersections of gender, race, and sexual orientation. We document increased risks of reporting an STI in a nationally representative sample of individuals with recent criminal legal involvement, particularly among white, heterosexual women; Black, heterosexual women; and white, gay/bisexual men. Interestingly, we did not find heightened risk of reporting an STI among Black, gay/bisexual men with recent criminal legal involvement, despite evidence documenting increased risk of STI/HIV among Black, gay/bisexual men [46,47]. One explanation could be that Black, gay/bisexual men just released from incarceration face unique individual and structural barriers, such as experiences of racism, that decrease their likelihood of obtaining STI testing [48]. Particularly, we found that healthcare utilization significantly predicted STI reporting in this sample, so it may be that Black gay/bisexual men are not able to engage with the healthcare system in ways that facilitate STI testing upon release from incarceration, which is particularly imperative since many STIs are asymptomatic.

This study, using an intersectional lens, also highlights several points of interventions for individuals with recent criminal legal involvement. First, substance use behaviors were consistently associated with increased risk of reporting a recent STI. A plausible mechanism for this relationship includes substance use leading to lower condom use and multiple partners, which is compounded by the increase in sexual risk behaviors and discordant partnerships of those recently leaving incarceration [49,50,51]. Particularly, our findings highlight unhealthy alcohol use behaviors, which are often overlooked, as consistently associated with increased STI risk among individuals with recent criminal justice involvement. A study of 530 women recently involved in the criminal legal system found that on days with alcohol use, the odds of condomless sex were 2.80 times higher (95% CI: 2.09–3.74) than on days without alcohol use [52]. More interventions may be warranted specifically targeting unhealthy alcohol consumption behaviors, alongside other substance use disorders, following incarceration. For example, a randomized trial of 100 individuals living with HIV and alcohol use disorders found that those prescribed naltrexone pre-release from incarceration for at least 12 weeks had significantly improved alcohol behaviors than those who received a placebo [53]. Our findings further validate the importance of current research, addressing substance use behaviors following incarceration with potential for reducing the burden of STIs [54,55,56].

Our findings are consistent with literature regarding sexual health and substance use behaviors among sexual minorities and by gender [57]. Structural inequities contribute to stereotypes that inform behaviors and discrimination—including homophobia and sexism—that place these groups in vulnerable positions. For example, experiences of discrimination have been shown to be associated with more substance use and sexual risk behaviors among sexual minority individuals [58,59]. Further, in the general population, Black, heterosexual women have the highest STI/HIV rates compared to heterosexual women from other racial groups [60]. Previous work has demonstrated that structural racism plays a key role in producing worse sexual health outcomes and substance use behaviors as well as inequitable access to treatment access among Black individuals [37,61,62,63,64,65]. Here, we find that white and Black, heterosexual women with recent criminal legal involvement also have elevated risk for reporting an STI, but that their risk of reporting an STI is diminished once overall substance use is controlled for. This finding suggests that substance use and sexual health interventions specifically designed for individuals with these lived experiences, such as Seeking Safety and the SISTA Project, could be particularly impactful for white and Black, heterosexual women leaving incarceration [66,67,68]. Further, addressing root causes of substance use, including poverty and trauma, and treatment post-release may be important in reducing STI risk in this population. Past work has highlighted that women often do not access substance use treatment post-release given barriers such as insurance coverage and financial insecurity, suggesting that such services should be easily accessible and guided by trauma-informed care [69,70,71]. Further, places that provide substance use treatment could be an avenue for integrating sexual health services, such as STI testing and access to pre-exposure prophylaxis (PrEP), for those leaving incarceration.

We also find that individual-level factors, such as substance use behaviors, may play a more limited role in explaining elevated STI risk among certain subpopulations. White, gay/bisexual men with recent criminal legal involvement were more likely to report a recent STI despite controlling for substance use behaviors. While access to substance use treatment remains important for white, gay/bisexual men leaving incarceration, other efforts such as educational programs focused on healthy sexual behaviors, HIV/STI screening interventions, and care continuum interventions to reduce sexual risk are needed for this subpopulation [72]. Our findings also suggest that additional research focused on contextual factors, including structural homophobia, may be warranted. A study of 450 young men who have sex with men found a primary syndemic construct of substance use behaviors, experiences of violence, and internalizing mental health conditions to be predictive of the number of condomless anal sex partners among white participants, but not among Latino or Black participants, suggesting the relationship between substance use and HIV/STI risk may operate uniquely for white sexual minority men [73]. Data from 174,209 men who have sex with men across Europe found that those living in countries with higher structural stigma against sexual minorities engage in riskier sexual behaviors, such as rarely using condoms, compared to those living in countries with lower structural stigma [74].

There are limitations to our study. Given the variables in NSDUH, we are unable to assess to what degree individuals are involved in the criminal legal system. Next, while we consider gender, race, and sexual orientation, there are other identities that may influence substance use and sexual behaviors post-release from incarceration, including age and socioeconomic class. Sample size and power issues prevent us from investigating the intersections of more than three identities, but additional research is needed to investigate further the influence of other subgroups of the identities included in our analysis (e.g., Native American) as well as other social identities (e.g., age). Relatedly, our ability to examine gender, race, and sexual orientation was restricted by the NSDUH survey questions, which had limited response options for assessing diverse gender identities (e.g., transgender) and sexual orientations (e.g., pansexuality). Notably, in our analysis, we grouped together lesbian and bisexual women with recent criminal legal involvement, due to sample size concerns, though it should be noted that these groups may engage in sexual acts that differ regarding risk, and future research is warranted, especially given that 33.3% of women within prisons identify as lesbian or bisexual [6]. Future work should consider further disaggregating sexual minority individuals into subpopulations. Respondents self-reported an STI diagnosis, which may be susceptible to bias, in lieu of diagnostic test results. We also excluded some respondents (less than 4%) due to missingness of variable information, which could lead to bias. Lastly, the NSDUH does not include individuals institutionalized within prisons, jails, nor individuals experiencing houselessness, a population heavily impacted by incarceration, which may underestimate the true associations.

## 5. Conclusions

Among those with recent criminal legal involvement, the relationship between substance use and STI risk varies along the intersections of gender, race, and sexual orientation. Findings from this study are meant to demonstrate the importance of targeting individuals with criminal justice involvement through an intersectional lens and understanding their needs to improve sexual health outcomes. Given our findings, intervention and prevention programming should target Black and white heterosexual women and white, gay, or bisexual, men following incarceration.

## Figures and Tables

**Table 1:** Sociodemographic Characteristics of a National Sample of Individuals with Criminal Legal Involvement in the Past 12 Months (N = 7260).

Characteristic	Percentage (95% Confidence Interval)
Age	
18–25	27.8 (26.4–29.1)
26–34	27.4 (26.0–28.7)
35–49	27.8 (26.4–29.2)
50–64	17.0 (15.3–18.7)
Gender	
Women	30.0 (28.4–31.5)
Men	70.0 (68.5–71.6)
Race and Ethnicity	
Hispanic	19.7 (18.0–21.5)
Non-Hispanic, Black	21.4 (20.0–22.8)
Non-Hispanic, white	51.9 (49.8–54.0)
Other	6.9 (6.1–7.8)
Sexual Orientation	
Heterosexual	91.4 (90.3–92.5)
Gay/Bisexual	8.6 (7.5–9.7)
Education	
Less than High School	27.2 (25.4–29.1)
High School	38.9 (37.1–40.6)
Some College	27.3 (25.9–28.7)
College	6.6 (5.8–7.3)
Employment	
Employed, Full-Time	46.9 (45.2–48.6)
Employed, Part-Time	11.7 (10.6–12.8)
Unemployed	14.3 (13.2–15.3)
Other	27.1 (25.7–28.5)
Poverty Status	
Living in Poverty	33.2 (31.4–35.0)
Income up to 2× Federal Poverty Level	27.2 (25.8–28.6)
Income More Than 2× the Federal Poverty Level	39.6 (37.8–41.4)
Metropolitan Status	
Large Metropolitan	50.0 (48.3–51.8)
Small Metropolitan	32.1 (30.5–33.7)
Nonmetropolitan	17.9 (16.6–19.1)
Marital Status	
Married	21.9 (20.5–23.4)
Widowed	1.8 (1.3–2.2)
Divorced or Separated	20.4 (18.8–22.1)
Never Married	55.9 (54.2–57.6)
Health Insurance	73.6 (72.5–74.7)
Health Care Utilization (mean visits, standard error)	2.9 (0.1)

**Table 2:** Association Between Substance Use and Sexually Transmitted Infection in the Past 12 Months Among a National Sample of Individuals with Recent Criminal Legal Involvement (N = 6986).

Substance Use Behaviors	Average Marginal Effect (Estimate; 95% Confidence Interval)
Overall Drug Use	
Any Illicit Drug Use	2.6% (1.3–4.0%) ***
Illicit Drug Use Disorder	3.0% (0.2–5.7 %) *
Illicit Drug or Alcohol Use Disorder	1.9 (−0.7–4.6%)
Polysubstance Use	3.5% (1.8–4.7%) ***
Alcohol	
Heavy Alcohol Use in Past 30 Days	2.4% (0.3–4.4%) *
Binge Alcohol Use in Past 30 Days	2.2% (0.8–3.7%) *
Alcohol Use Disorder	2.8% (0.2–5.5%) *
Marijuana	
Any Marijuana Use	3.0% (1.6–4.3%) ***
Marijuana Use Disorder	2.1% (−3.0–7.2%)
Cocaine	
Any Cocaine Use	3.2% (0.6–5.7%) *
Cocaine Use Disorder	9.6 (−0.4–19.6)
Crack	
Any Crack Use	−0.8% (−3.5–2.0%)
Heroin	
Any Heroin Use	3.4% (−0.6–7.4%)
Heroin Use Disorder	17.2 (−0.9–43.4%)
Hallucinogens	
Any Hallucinogens Use	4.1% (0.8–7.4%) *
Hallucinogen Use Disorder	8.7 (−2.7–20.1%)
Inhalants	
Any Inhalant Use	7.1% (−1.2–15.4%)
Inhalant Use Disorder	8.0% (−10.7–26.8%)
Methamphetamine	
Any Methamphetamine Use	3.7% (0.7–6.9%) *
Methamphetamine Use Disorder	−1.9% (−7.6–3.8%)

* ≤ 0.05; *** ≤ 0.001.

**Table 3:** Role of Gender, Race, and Sexual Orientation in the Association between Overall Drug Use Behaviors and Sexually-Transmitted Infection among a National Sample of Individuals with Recent Criminal Legal Involvement (N = 4530) ^1^.

		Average Marginal Effect (Estimate; 95% Confidence Interval)		Average Marginal Effect (Estimate; 95% Confidence Interval)				Average Marginal Effect (Estimate; 95% Confidence Interval)
	Any Illicit Drug Use	2.6% (0.9–4.3%) **	Illicit Drug Use Disorder	3.3% (−0.8−7.4 %)	Illicit Drug Use Disorder or Alcohol Use Disorder	1.8% (−0.8–4.5%)	Polysubstance Use	3.5% (2.0–5.0%) ***
White Heterosexual Men		Ref.		Ref.		Ref.		Ref.
White Heterosexual Women		1.2% (−0.9–3.4%)		1.3% (−0.9–3.4%)		1.3% (−0.8−3.4%)		1.4% (−0.8–3.5%)
Black Heterosexual Women		3.3% (−0.5–7.0%)		3.1% (−0.6–6.9%)		3.2% (−0.6–6.9%)		3.3% (−0.5–7.0%)
White GayBbisexual Men		16.2 % (2.3–30.1%) *		16.7% (2.9–30.5%) *		16.8% (2.9–30.7%) *		16.0% (2.1–29.9%) *
Age		−0.6 % (−1.0−0.3%) ***		−0.7% (−1.0–−0.4%) ***		−0.7% (−1.0–−0.4%) ***		−0.6% (−0.9–−0.3%)
Metropolitan Status		−0.0 % (−0.8–0.8%)		−0.0% (−0.9–0.7%)		−0.1% (−0.9–0.7%)		−0.0% (−0.8–0.8%)
Education Level		0.1 % (−0.4–0.5%)		0.0% (−0.4–0.5%)		0.1% (−0.4–0.5%)		0.1% (−0.4–0.5%)
Marital Status		−0.2% (−0.8–0.5%)		−0.1% (−0.7−0.6%)		−0.1% (−0.7–0.6%)		−0.2% (−0.9–0.4%)
Healthcare Utilization		0.3% (0.1−0.6%) *		0.3% (0.1−0.6%) *		0.3% (0.1–0.6%) *		0.3% (0.0–0.6%) *
Poverty Status		−0.1% (−1.1–0.8)		−0.2% (−1.1 −0.7%)		−0.1 (−0.3–0.1)		−0.2% (−1.1–0.8%)

* ≤ 0.05; ** ≤ 0.01; *** ≤ 0.001. ^1^ In our model, we included our four primary substance use variables (any illicit drug use, illicit drug use disorder, illicit drug use disorder or alcohol use disorder, and polysubstance use) as covariates in each of the four regressions alongside age, metropolitan status, education level, marital status, healthcare utilization, and poverty status. Our outcome remained self-reported STI in the past 12 months while our variable of interest was the intersectional social groups (i.e., white, heterosexual men; white heterosexual women; Black heterosexual women; and white gay/bisexual men).

## Data Availability

Publicly available date from the National Survey on Drug Use and Health can be accessed at the following: National Survey on Drug Use and Health (rti.org), last accessed on 30 December 2021.

## Abbreviations

DSM IV	Diagnostic and Statistical Manual of Mental Disorders, 4th edition
HIV	Human immunodeficiency virus
NSDUH	National Survey on Drug Use and Health
PrEP	Pre-exposure prophylaxis
STI	Sexually transmitted infection
U.S.	United States
